# Supplementary material for: Dissection of genetic variation and evidence for pleiotropy in male pattern baldness
Source: Nat Commun. 2018 Dec 20;9:5407. doi: 10.1038/s41467-018-07862-y (PMC6302097; doi:10.1038/s41467-018-07862-y)
Supplement: Supplementary file 1 — Supplementary Information [file 41467_2018_7862_MOESM1_ESM.pdf]

Supplementary Information

Dissection of genetic variation and evidence for pleiotropy for male pattern baldness

Chloe X Yap[1], Julia Sidorenko[1,2], Yang Wu[1], Kathryn E Kemper[1], Jian Yang[1,3],
Naomi R Wray[1,3], Matthew R Robinson[1,4,5], Peter M Visscher[1,3]

[1]Institute for Molecular Bioscience, University of Queensland, Queensland QLD 4072, Australia
[2]Estonian Genome Center, Institute of Genomics, University of Tartu, 51010 Tartu, Estonia
[3]Queensland Brain Institute, University of Queensland, Queensland QLD 4072, Australia
[4]Department of Computational Biology, University of Lausanne, 1015 Lausanne, Switzerland
[5]Swiss Institute of Bioinformatics, CH-1015 Lausanne, Switzerland
*corresponding author peter.visscher@uq.edu.au

MPB phenotype data collection

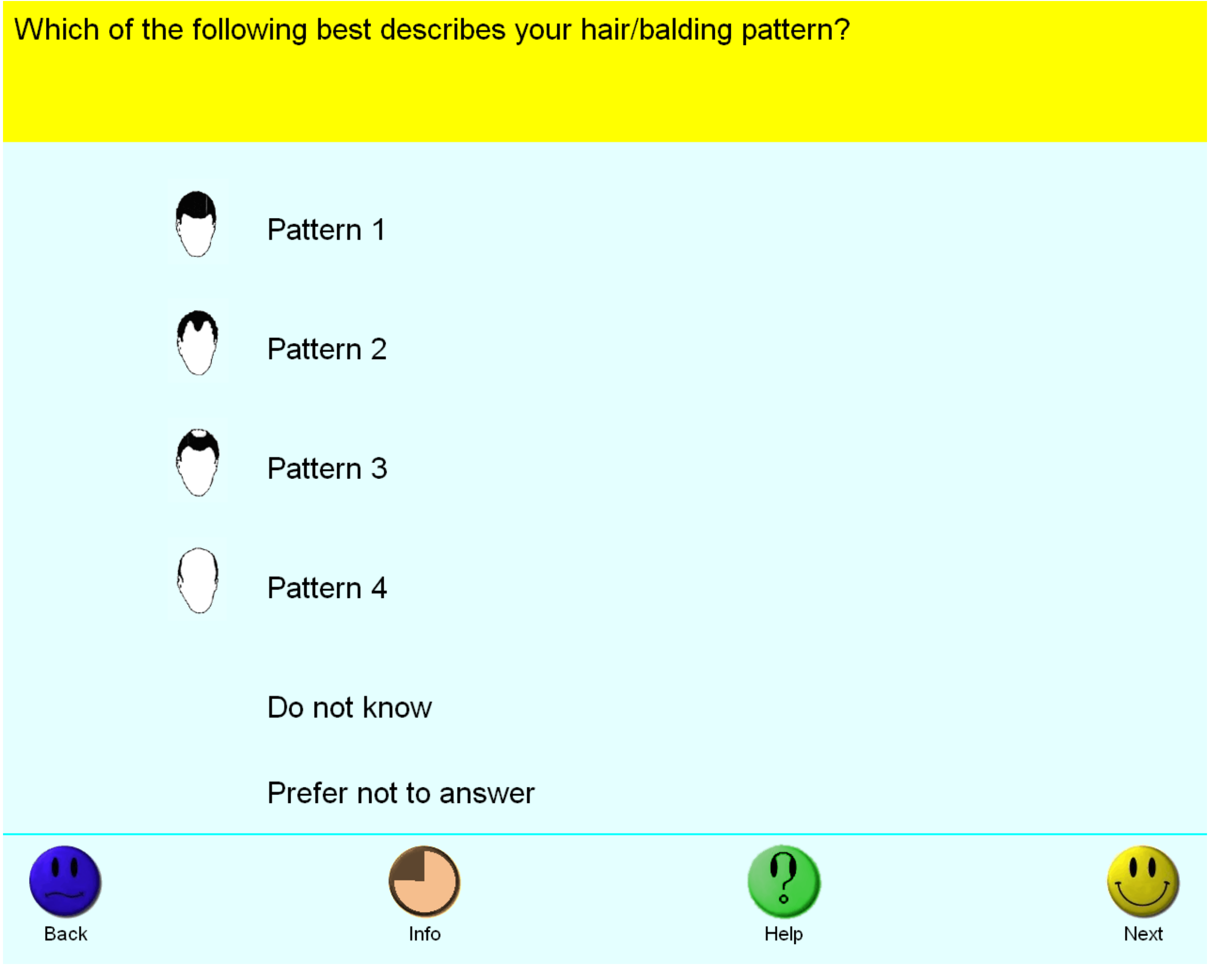

Supplementary Figure 1: Screenshot of the MPB survey question provided to UK Biobank participants. Image provided courtesy of the UK Biobank, and is available at [https://biobank.ctsu.ox.ac.uk/crystal/refer.cgi?id=100423].

.

Data QC flow diagram

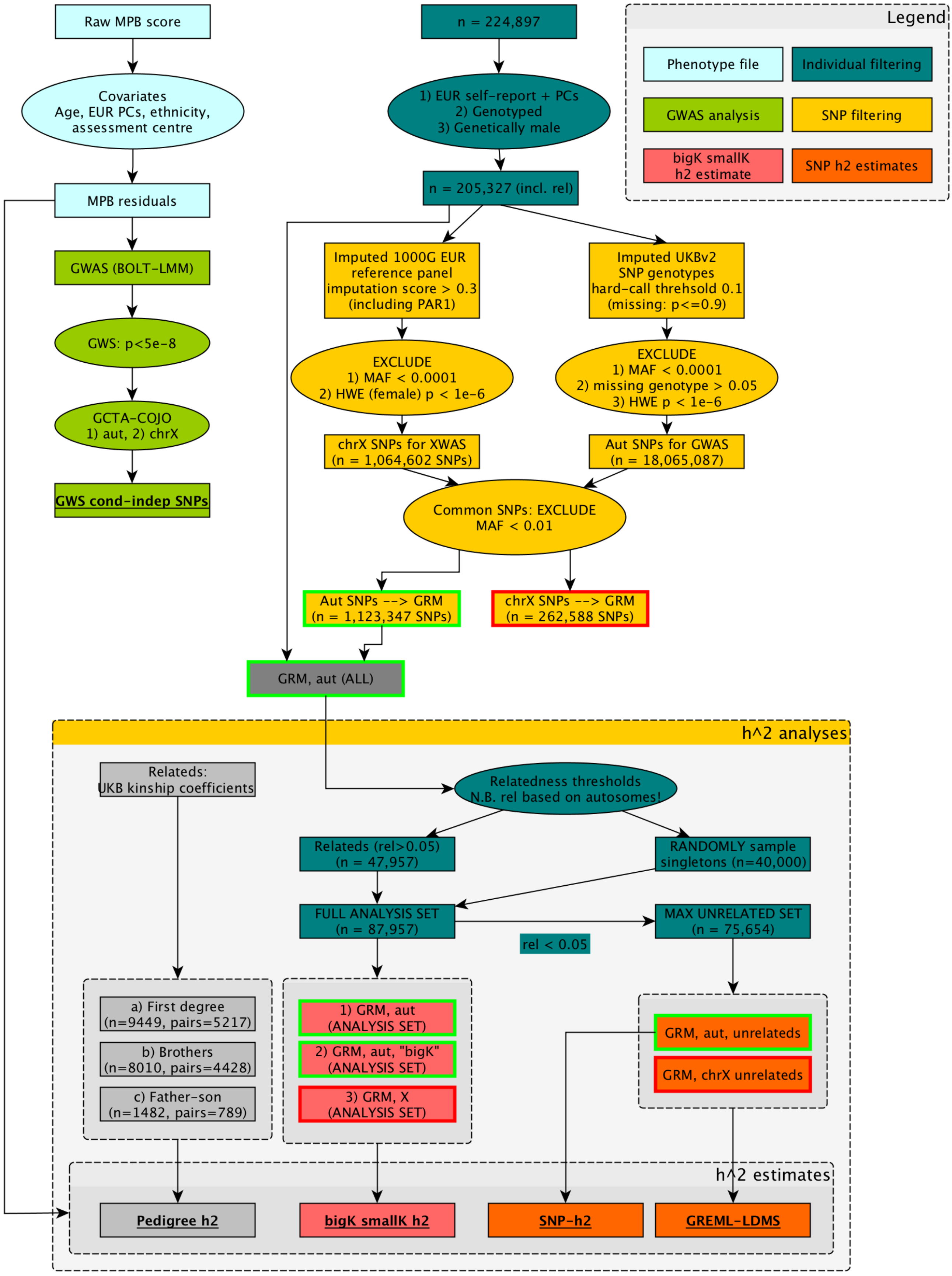

Supplementary Figure 2: Flow diagram showing data QC steps and an overview of analyses. Green outline denotes GRMs derived from autosomal data versus red outline denotes those derived from X-chromosome data.

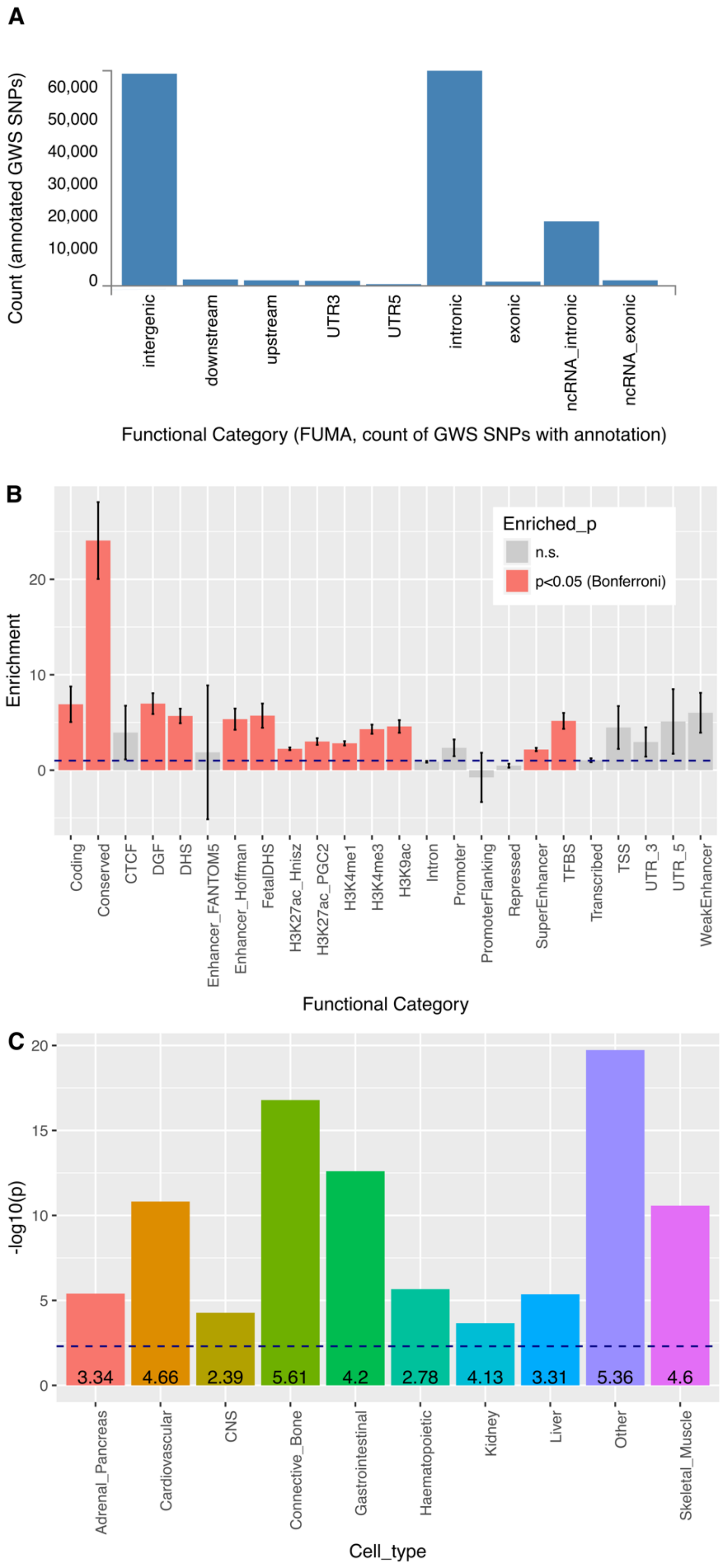

Supplementary Figure 3: Cellular elements in MPB. (A) Counts of GWS SNPs under the BOLT-LMM non-infinitesimal mixed model association test (including SNPs in LD with lead SNPs) with ANNOVAR functional annotations. Output from FUMA[1]. (B) Heritability partitioning by functional annotation using LDSC[2]. Enrichment within a category is defined as the proportion of h^2_{SNP} divided by the proportion of SNPs. Analyses included SNPs within a 500bp window around the annotated site, as described by Finucane et al 2015. Blue horizontal broken line with intercept=1 denotes no enrichment. Red bars denote functional categories where the enrichment *P*<0.05 (Bonferroni-corrected). Error bars denote standard error. (C) Plot showing –log10(p) for enrichment, per cell-type-group. Blue dotted line denotes the Bonferroni-corrected threshold. Numbers denote the value for enrichment.

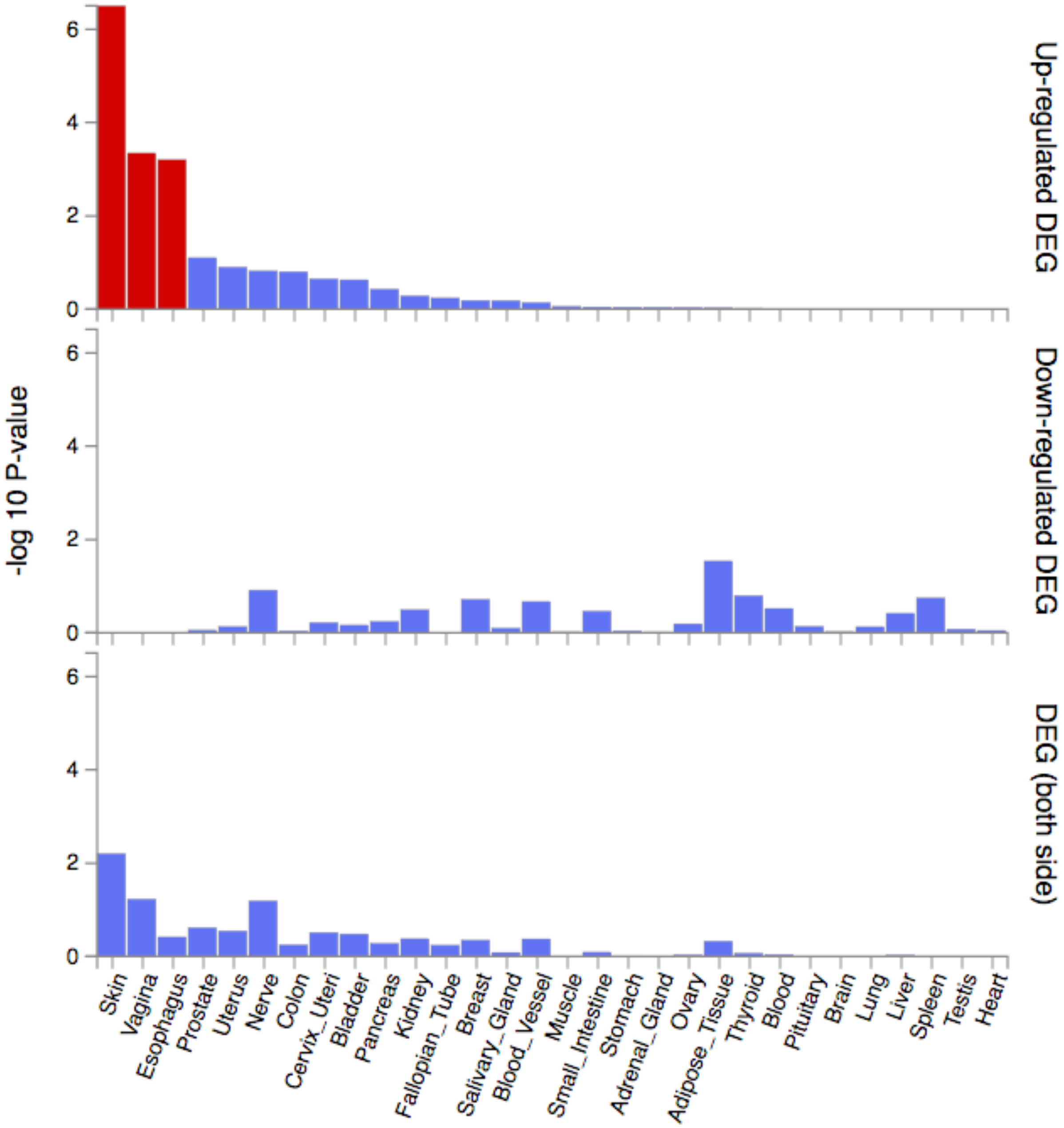

Supplementary Figure 4: FUMA[1] GENE2FUNC differentially-expressed genes (DEG) output drawing upon GTEx v6 data for 30 general tissue types. Red bars denote significantly enriched DEG sets (*P*<0.05, Bonferroni-corrected).

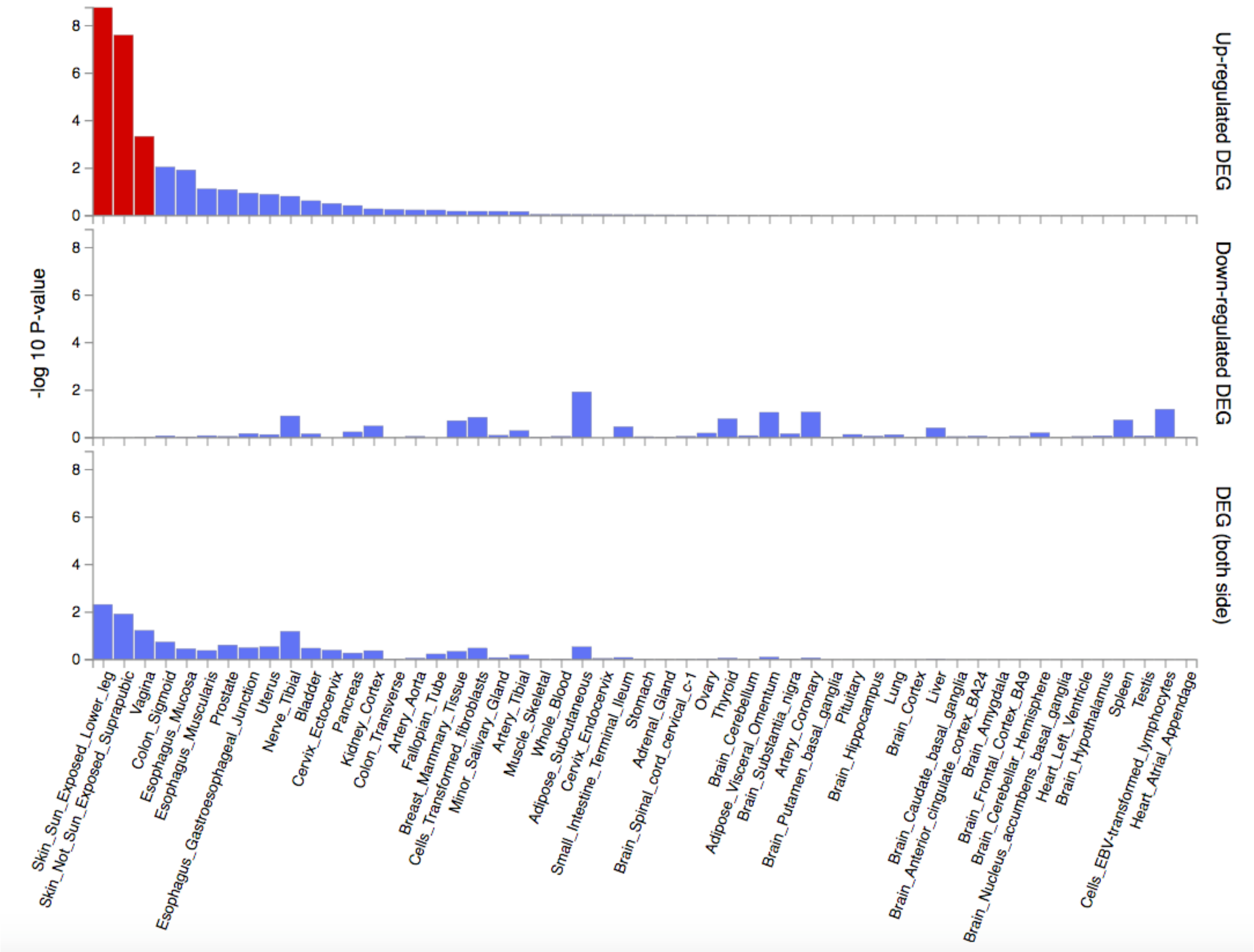

Supplementary Figure 5: FUMA[1] GENE2FUNC differentially-expressed genes (DEG) output drawing upon GTEx v6 data for 53 tissue types. Red bars denote significantly enriched DEG sets (*P*<0.05, Bonferroni-corrected).

Supplementary Note 1: Further discussion on interesting and plausible biological pathways

X-chromosome annotations

We focused particularly on the X-chromosome as it has a known, prominent signal[3-11]. The GWS loci (identified through conditional analysis to assess independence) covered eight chromosome bands. 13 of the 26 X-chromosome loci (Fig.3c green points, Fig.3d) were located within a ~2.3 Mb window, in the Xq12 band, between the genes *SPIN4/ARHGEF9, MSN/VSIG4, HEPH/EDA2R*, *EDA2R/AR*, *AR/OPHN1*, *YIPF6/STARD8* and within the *OPHN* gene (Supplementary Data 5). An additional seven loci were located within the previously-identified Xp11.21 band[12] between *PAGE2/FAM104B*, *PAGE3/MAGEH1*, *MAGEH1/USP51*, *KLF8/UBQLN2* and *UBQLN2/SPIN3* (Supplementary Data 5). The other identified loci occurred between *MXRA*5 and *PRKX* (Xp22.3); between *FAM9*A and *FAM9B* (Xp22.31); between *ZXDA* and the centromere (Xp11.1), within *EDA* (Xq13.1), between *FTX/SLC16A2* (Xq13.2) and within *BGN1* (Xq28) (Supplementary Data 5). In comparison, Heilmann-Heimbach's analysis identified three distinct loci on the X-chromosome[12].

Downstream GWAS results (FUMA results)

The results of our downstream GWAS investigations were consistent with the dynamic nature of hair biology. Hair follicles cycle through the stages of resting (telogen), growth (anagen) and regression (catagen), regulated by transcriptional changes in the dermal papilla. Concordant with this dynamic regulation, the vast majority of SNPs were located in intergenic and intronic regions (Supplementary Fig.3a), and MAGMA competitive gene set analysis[1] also showed enrichment for genes related to transcriptional elements (Supplementary Data 13, Supplementary Data 14). In addition, the partitioned heritability analysis[2] in the baseline model was particularly enriched for histone modifications (H3K27ac, H3K4me1, H3K4me3, H3K9ac) (Supplementary Fig.3b).

The downstream GWAS analyses are also consistent with the tissue types involved in hair development. Follicle development involves interactions between the apposed epithelium and mesenchyme (later becoming the dermis), and our analyses implicated mesenchymal and epidermal gene sets. Specifically, the dermal papilla cell population that regulates hair follicle growth is of mesenchymal origin[13] and undergoes transcriptional signalling changes affecting β-catenin signalling and the FGF pathway[13]. The relevance of these pathways was supported by FUMA GENE2FUNC analysis[1] which implicated the Wnt/β-catenin signalling and the UV response pathways, within the

Hallmark gene sets. This corresponds with *in vitro* experiments in epidermal stem cells which have shown that activation of the androgen receptor (*AR*) suppresses the Wnt/β-catenin pathway, and in turn promotes hair follicle and sebaceous gland development[14,15]. The partitioned heritability analysis (by cell-type group) also supported the MAGMA gene-set analysis results, finding that enrichment was strongest in the mesenchyme-derived connective tissue and bone cell-group-type, as well as in the category classified as "Other" (adipose nuclei, breast tissue, ovary, penis foreskin and placenta) (Supplementary Fig.3c).

Downstream GWAS results (partitioned heritability by cell-type-specific elements)

We also used the LDSC partitioned heritability analysis[2] to investigate whether cell-type-specific elements were enriched for MPB h^2_{SNP} for ten tissue types. This analysis found that all of the ten tissues were enriched (P<0.005, Bonferroni-corrected for ten tissue types), of which the connective tissue and bone, and "Other" (which includes adipose nuclei, breast tissue, ovary, penis foreskin and placenta) categories had particularly strong associations (Supplementary Fig.3c, Supplementary Data 15). The connective tissue and bone category was most obviously relevant to MPB, and a partitioned h^2_{SNP} analysis within this tissue type showed a similar h^2 distribution as that for the baseline model.

Pleiotropy inferences: potential gene-level evidence

The pleiotropy implied by the whole-genome genetic correlation analyses also appears to have a basis among the COJO GWS SNPs, as shown by the ICSNPathway[16] analysis. We performed GeneCards lookups of genes that were nearby the 623 COJO GWS SNPs that had been grouped within the "Reproductive" and "Reproductive Pathways" ontologies. Several were related to mesenchymal, androgen, and sex development pathways. For example, *TGFB1*, *SMAD3* and *BMP4* are important in the TGF-β pathway, which interacts with androgen signalling. In prostate cancer, TGF-β and *SMAD3* enhance *AR*-mediated transactivation[17], and *BMP* mediates androgen-stimulated prostate cell growth[18]. With respect to the genetic correlation with early puberty onset, *CRHR1* encodes the corticotrophin releasing hormone receptor, which regulates the HPA axis, and therefore relates to adrenarche. *CRHR1* is in the vicinity of the GWS SNP rs199441 on chromosome 17 which has a large effect size (beta=-0.11, P=1.60e-197, Supplementary Data 4). For bone mineral density, *PTHLH* codes for parathyroid hormone-like protein; parathyroid

hormone is the major regulator of plasma calcium levels, and maintains homeostasis by reducing bone mineral density. The *TGF-β* pathway (which also involves *Wnt* proteins) is also pleiotropic in bone development.

Fertility inferences: potential gene-level evidence

Other genes that were identified by ICSNPathway[16] within the "Reproductive" and "Reproductive Processes" gene sets also implicate sex development and fertility. For males, *CCNA1* controls germline meiosis in the testes, *SPAG1* encodes sperm associated antigen 1 and is associated with infertility, *CDYL* encodes chromodomain Y-like protein which is an autosomal gene also linked to infertility, and *NME5* contributes to spermiogenesis. For female fertility, *BMP15* is also related to the *TGF-β* pathway and contributes to oocyte maturation, *FOXL2* (associated with ovarian development) and *TRO* is related to placental development. *AMHR2* encodes anti-Mullerian hormone receptor type 2 which is critical for male sex differentiation (along with testosterone), and oocyte development and interacts with the *TG*F-β signalling pathways.

Future traits of interest to study

There are other traits that should be investigated in relation to MPB. The relationship between MPB and polycystic ovary syndrome (PCOS) is worth studying as a female-limited proxy trait[19,20]. Small pedigree studies (n=14) have suggested that male relatives of PCOS sufferers have an increased risk of early onset MPB, irrespective of ethnicity[19]. PCOS is a hyperandrogenetic syndrome[21] and patients present with a combination of infertility, hirsutism, acne, menstrual changes and metabolic syndrome[19,21]. Although PCOS is a well-defined clinical syndrome that only affects females, it has recently been suggested that there may be a male equivalent characterised by early-onset MPB and metabolic syndrome[22]. Unfortunately, PCOS data are only available for 52 women in the UKB dataset, and to our knowledge, there are no publically-available GWAS summary statistics for this trait. Bone development is another potential trait of interest as bone age is used in clinical paediatrics as a check for age to investigate precocious[23] or delayed puberty[24]. This may suggest an association with puberty-onset[25] that is worth studying in future. In keeping with the theme of developmentally-related traits in mesenchymal tissue, a third trait of interest could be odontogenesis. This development pathway was also flagged in the FUMA analysis, and permanent tooth eruption occurs during development and puberty; however, previous studies have only shown a weak relationship between tooth eruption and pubertal growth spurt[25,26].

Supplementary Note 2: Comparison of effect sizes for MPB and 2014 Wood et al. Height GWAS

Methods

We applied a z-score transformation to beta values calculated from the top 705 SNPs in the present MPB GWAS, and compared the effect size distribution to the top 697 SNPs identified in the 2014 Wood et al. height GWAS (Supplementary Data 4, height GWAS https://www.nature.com/articles/ng.3097#supplementary-information). For the MPB data, we used the conditionally-independent GWS SNPs identified in the autosomal subset as the height GWAS was analysed autosomal data. These studies are similar in that they are highly-powered (n_MPB = 205,327; n_Height = 253,288) and with respect to number of GWS hits (n_MPB = 597, though we used the top 705 here; n_Height = 697). Volcano plots and a histogram (using absolute z-score) were generated of z-scores (Supplementary Fig.6).

Results and Discussion

MPB appeared to have a greater number of larger beta coefficients than height (Supplementary Fig.6), yet also had more variants with small effect sizes at the GWS level. Under an infinitesimal model, this may suggest that MPB has a more oligogenic architecture than height; that is, a smaller set of SNPs drives more of genetic variation. Notably however, the X-chromosome was not included in this analysis to allow a fairer comparison, though this means that the prominent X-chromosome effect observed in MPB was not captured.

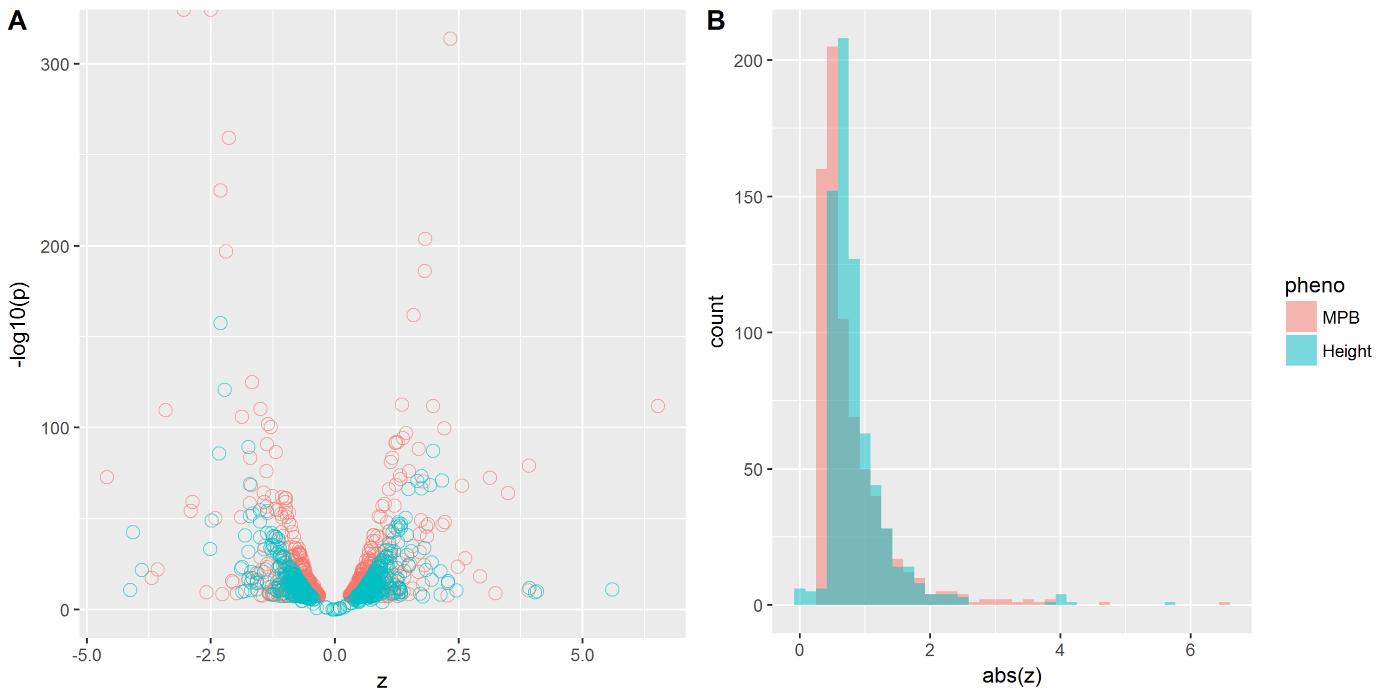

Supplementary Figure 6: Comparison of *Z*-scores calculated from conditionally-independent GWS SNPs MPB (pink, n=705) and height (green, n=697). A) Volcano plot of z-scores. B) Histogram of absolute z-scores.

References

1 Watanabe, K., Taskesen, E., van Bochoven, A. & Posthuma, D. Functional mapping and annotation of genetic associations with FUMA. *Nat Commun* **8**, 1826, doi:10.1038/s41467-017-01261-5 (2017).

2 Finucane, H. K. *et al.* Partitioning heritability by functional annotation using genome-wide association summary statistics. *Nat Genet* **47**, 1228-1235, doi:10.1038/ng.3404 (2015).

3 Hillmer, A. M. *et al.* Susceptibility variants for male-pattern baldness on chromosome 20p11. *Nat Genet* **40**, 1279-1281, doi:10.1038/ng.228 (2008).

4 Richards, J. B. *et al.* Male-pattern baldness susceptibility locus at 20p11. *Nat Genet* **40**, 1282-1284, doi:10.1038/ng.255 (2008).

5 Ellis, J. A., Stebbing, M. & Harrap, S. B. Polymorphism of the androgen receptor gene is associated with male pattern baldness. *J Invest Dermatol* **116**, 452-455, doi:10.1046/j.1523-1747.2001.01261.x (2001).

6 Hayes, V. M. *et al.* The E211 G>A androgen receptor polymorphism is associated with a decreased risk of metastatic prostate cancer and androgenetic alopecia. *Cancer Epidemiol Biomarkers Prev* **14**, 993-996, doi:10.1158/1055-9965.EPI-04-0778 (2005).

7 Hillmer, A. M. *et al.* Genetic variation in the human androgen receptor gene is the major determinant of common early-onset androgenetic alopecia. *Am J Hum Genet* **77**, 140-148, doi:10.1086/431425 (2005).

8 Prodi, D. A. *et al.* EDA2R is associated with androgenetic alopecia. *J Invest Dermatol* **128**, 2268-2270, doi:10.1038/jid.2008.60 (2008).

9 Cobb, J. E., Zaloumis, S. G., Scurrah, K. J., Harrap, S. B. & Ellis, J. A. Evidence for two independent functional variants for androgenetic alopecia around the androgen receptor gene. *Exp Dermatol* **19**, 1026-1028, doi:10.1111/j.1600-0625.2010.01132.x (2010).

10 Heilmann, S. *et al.* Androgenetic alopecia: identification of four genetic risk loci and evidence for the contribution of WNT signaling to its etiology. *J Invest Dermatol* **133**, 1489-1496, doi:10.1038/jid.2013.43 (2013).

11 Hagenaars, S. P. *et al.* Genetic prediction of male pattern baldness. *PLoS Genet* **13**, e1006594, doi:10.1371/journal.pgen.1006594 (2017).

12 Heilmann-Heimbach, S. *et al.* Meta-analysis identifies novel risk loci and yields systematic insights into the biology of male-pattern baldness. *Nat Commun* **8**, 14694, doi:10.1038/ncomms14694 (2017).

13 Driskell, R. R., Clavel, C., Rendl, M. & Watt, F. M. Hair follicle dermal papilla cells at a glance. *J Cell Sci* **124**, 1179-1182, doi:10.1242/jcs.082446 (2011).

14 Kretzschmar, K., Cottle, D. L., Schweiger, P. J. & Watt, F. M. The Androgen Receptor Antagonizes Wnt/beta-Catenin Signaling in Epidermal Stem Cells. *J Invest Dermatol* **135**, 2753-2763, doi:10.1038/jid.2015.242 (2015).

15 Inui, S. & Itami, S. Androgen actions on the human hair follicle: perspectives. *Exp Dermatol* **22**, 168-171, doi:10.1111/exd.12024 (2013).

16 Zhang, K. *et al.* ICSNPathway: identify candidate causal SNPs and pathways from genome-wide association study by one analytical framework. *Nucleic Acids Res* **39**, W437-443, doi:10.1093/nar/gkr391 (2011).

17 Kang, H. Y. *et al.* From transforming growth factor-beta signaling to androgen action: Identification of Smad3 as an androgen receptor coregulator in prostate cancer cells. *P Natl Acad Sci USA* **98**, 3018-3023, doi:DOI 10.1073/pnas.061305498 (2001).

18 Guo, X. & Wang, X. F. Signaling cross-talk between TGF-beta/BMP and other pathways. *Cell Res* **19**, 71-88, doi:10.1038/cr.2008.302 (2009).

19 Carey, A. H. *et al.* Polycystic ovaries and premature male pattern baldness are associated with one allele of the steroid metabolism gene CYP17. *Hum Mol Genet* **3**, 1873-1876 (1994).

20 Carey, A. H. *et al.* Evidence for a single gene effect causing polycystic ovaries and male pattern baldness. *Clin Endocrinol (Oxf)* **38**, 653-658 (1993).

21 Azziz, R. *et al.* Positions statement: criteria for defining polycystic ovary syndrome as a predominantly hyperandrogenic syndrome: an Androgen Excess Society guideline. *J Clin Endocrinol Metab* **91**, 4237-4245, doi:10.1210/jc.2006-0178 (2006).

22 Cannarella, R., Condorelli, R., Mongioi, L. M., La Vignera, S. & Calogero, A. Does a male polycystic ovarian syndrome equivalent exist? *Journal of Endocrinological Investigation* **41**, 49-57, doi:10.1007/s40618-017-0728-5 (2018).

23 Carel, J. C., Lahlou, N., Roger, M. & Chaussain, J. L. Precocious puberty and statural growth. *Hum Reprod Update* **10**, 135-147, doi:10.1093/humupd/dmh012 (2004).

24 Cooke, D. W., Divall, S. A. & Radovick, S. in *Williams Textbook of Endocrinology (Thirteenth Edition)* (eds Shlomo Melmed, Kenneth S. Polonsky, P. Reed Larsen, & Henry M. Kronenberg) 964-1073 (Content Repository Only!, 2016).

25 Demirjian, A., Buschang, P. H., Tanguay, R. & Patterson, D. K. Interrelationships among measures of somatic, skeletal, dental, and sexual maturity. *American Journal of Orthodontics and Dentofacial Orthopedics* **88**, 433-438, doi:10.1016/0002-9416(85)90070-3 (1985).

26 Hagg, U. & Taranger, J. Dental emergence stages and the pubertal growth spurt. *Acta Odontol Scand* **39**, 295-306 (1981).
